# A cryo-TSEM with temperature cycling capability allows deep sublimation of ice to uncover fine structures in thick cells

**DOI:** 10.1038/s41598-021-00979-z

**Published:** 2021-11-01

**Authors:** Jiro Usukura, Akihiro Narita, Tomoharu Matsumoto, Eiji Usukura, Takeshi Sunaoshi, Syunya Watanabe, Yusuke Tamba, Yasuhira Nagakubo, Takashi Mizuo, Junzo Azuma, Masako Osumi, Kazutaka Nimura, Ryuichiro Tamochi, Yoichi Ose

**Affiliations:** 1 Facility of Ultra High Voltage Electron Microscope, Institute of Materials and Systems for Sustainability, Nagoya University, Furo-cho, Nagoya, 464-8601 Japan; 2 Graduate School of Science, Nagoya University, Nagoya, 464-8601 Japan; 3 Graduate School of Medicine, Kyoto University, Kyoto, 606-8303 Japan; 4 Hitachi High-Tech Corporation, Tokyo, 105-6409 Japan; 5 Japan Women’s University, Tokyo, 112-8681 Japan

**Keywords:** Cryoelectron microscopy, Cytoskeleton, Actin, Microtubules

## Abstract

The scanning electron microscope (SEM) has been reassembled into a new type of cryo-electron microscope (cryo-TSEM) by installing a new cryo-transfer holder and anti-contamination trap, which allowed simultaneous acquisition of both transmission images (STEM images) and surface images (SEM images) in the frozen state. The ultimate temperatures of the holder and the trap reached − 190 °C and − 210 °C, respectively, by applying a liquid nitrogen slush. The STEM images at 30 kV were comparable to, or superior to, the images acquired with conventional transmission electron microscope (100 kV TEM) in contrast and sharpness. The unroofing method was used to observe membrane cytoskeletons instead of the frozen section and the FIB methods. Deep sublimation of ice surrounding unroofed cells by regulating temperature enabled to emerge intracellular fine structures in thick frozen cells. Hence, fine structures in the vicinity of the cell membrane such as the cytoskeleton, polyribosome chains and endoplasmic reticulum (ER) became visible. The ER was distributed as a wide, flat structure beneath the cell membrane, forming a large spatial network with tubular ER.

## Introduction

Cryo-transmission electron microscopy (cryo-TEM) has become widespread, and several dedicated commercial microscopes are currently sold. In the past, cryo-transfer holders were sold separately, and were used in conjunction with general-purpose TEMs, because commercial TEMs designed for frozen samples were rarely available. The major differences between these types of instruments lies in how the sample is brought into the electron microscope. Currently, frozen samples can be placed in a cassette-type holder and moved in and out of the microscope with an integrated autoloader. In this case, temperature cycling is limited compared to that with a side-entry cryotransfer holder. Therefore, it is difficult for a cassette-type holder to reduce the ice-layer thickness by quickly raising the specimen temperature during imaging. Thus, recent cryo-TEMs are suitable for imaging protein molecules embedded in a thin layer of ice, rather than frozen cells containing large amounts of ice. Because of necessity and urgency, the structural analyses of proteins and viruses, are much more common than those of cells. Other factors include the ease of quick vitreous-ice freezing of molecular-sized specimens and the development of high-sensitivity direct-detection cameras. Imaging at low electron doses with a high-sensitivity camera, as well as significant suppression of molecular deformation by vitreous ice freezing, has maximized the ability of single-particle analysis. The resolution of molecular shapes reconstructed by single-particle analysis, combined with cryo-TEMs, almost approaches the atomic level of X-ray crystallography, despite dealing with dispersed proteins^1–5^. Although cryo-electron microscopy is often used for analyzing molecular structures at atomic resolution, it can also be used to image unfixed, native cell structures. Despite it may not be always certain that cells can be really frozen without incurring formation of ice crystals due to its bulky nature, it is essential to observe frozen cells to have a glimpse of authentic intracellular fine structures within. Recent reports on quickly frozen cells have described high-resolution intracellular fine structures such as desmosomes, ribosomes, and nuclear lamina, which have never been imaged with a standard TEM^6–23^. Furthermore, via tomography, the molecular structure of the membrane and several types of filaments have been analysed with high, three-dimensional, resolution^7–22^. Though the resolution is clearly lower than that of single particle analysis, the observations at the level of organelle do not necessarily require atomic resolution. Instead, image contrast is more important than resolution. Because the ice thickness varies from cell to cell, it is necessary to reduce it via temperature regulation. The cryo-transmission scanning electron microscope (cryo-TSEM) reported here was developed with those considerations. It is based on a 30-kV acceleration voltage, in-lens type, scanning electron microscope (SEM, Hitch SU9000 Tokyo, Japan), equipped with a detector for transmitted electrons and a cold-field emission gun (CFEG). Therefore, this cryo-TSEM enables simultaneous acquisition of a scanning transmission electron (STEM) image and a secondary electron (SEM) image. Furthermore, a side-entry cryotransfer holder was developed to control the sample temperature in fine steps. In this cryo-TSEM, transmission electron images are formed with amplitude contrast obtained by scanning a thin electron beam in a point-by-point manner, which is peculiar to the STEM optical system. A clear focussed image can be obtained, without phase contrast via defocusing. Therefore, for single-particle analysis, it has the advantage that correction of the contrast transfer function (CTF) is unnecessary^24^. For several years, Elbaum et al. have been using 200 kV cryo-STEM for the tomography of biological samples^24–26^. They showed that cryo-STEM was effective for biological imaging, while explaining the physical characteristics of cryo-tomography for bacteria and cells^24–26^. The single-particle analysis and tomography cited herein were obtained using cryo-TEM or cryo-STEM with medium acceleration voltages of 200 kV or 300 kV that offer the required length of electron mean free path. The mean free path in 300-kV TEM is about 300 nm in liquid water and longer in biological samples^27,28^. This parameter is important, particularly for tomography, because the sample tilt would increase the sample thickness. As the trend of recent cryo-EM development appears to exploit a high acceleration voltage to observe samples embedded in thick ice directly without reducing the ice layer, our development seems to be against such a trend. However, since our goal here was to develop a compact cryo-electron microscope system easier to use for frozen cells with 1-nm resolution, we gratifyingly deliver those capabilities based on a 30-kV cryo-TSEM.

## Results

### Construction of cryo-TSEM

**Figure 1:**
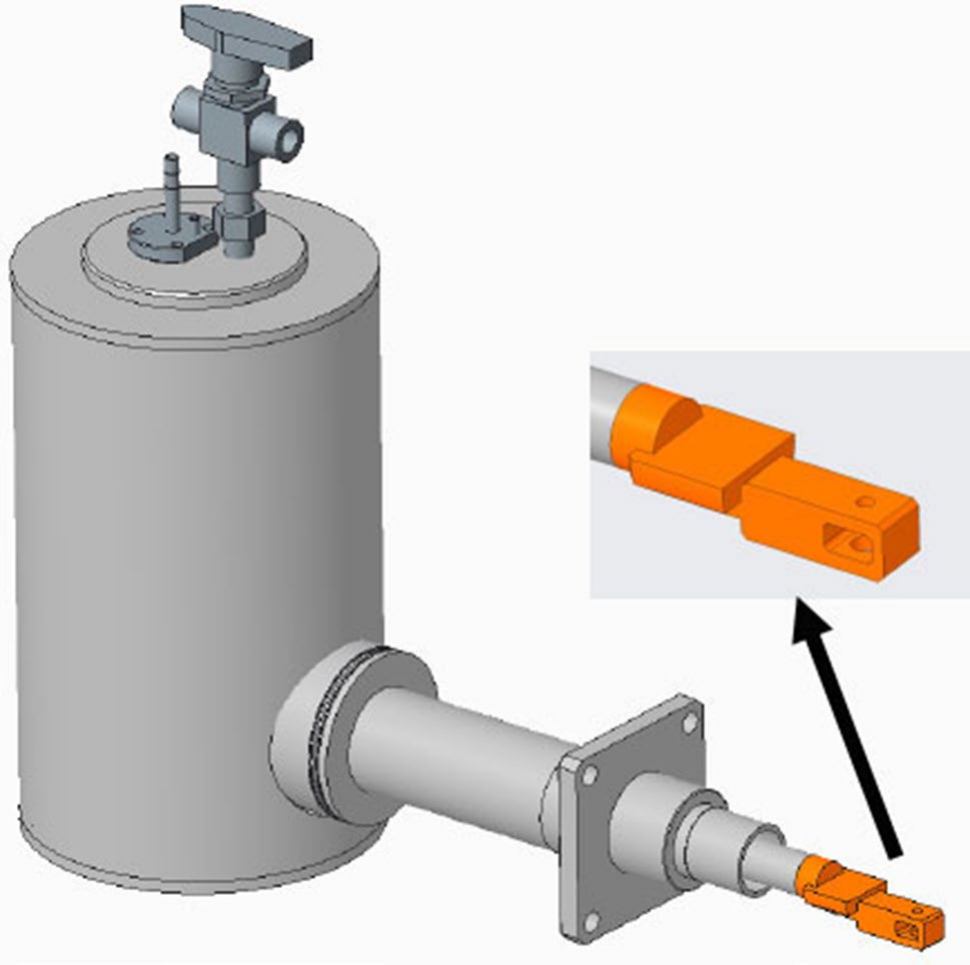
Schematic of the anti-contamination trap. The inset shows an enlarged view of the tip.

**Figure 2:**
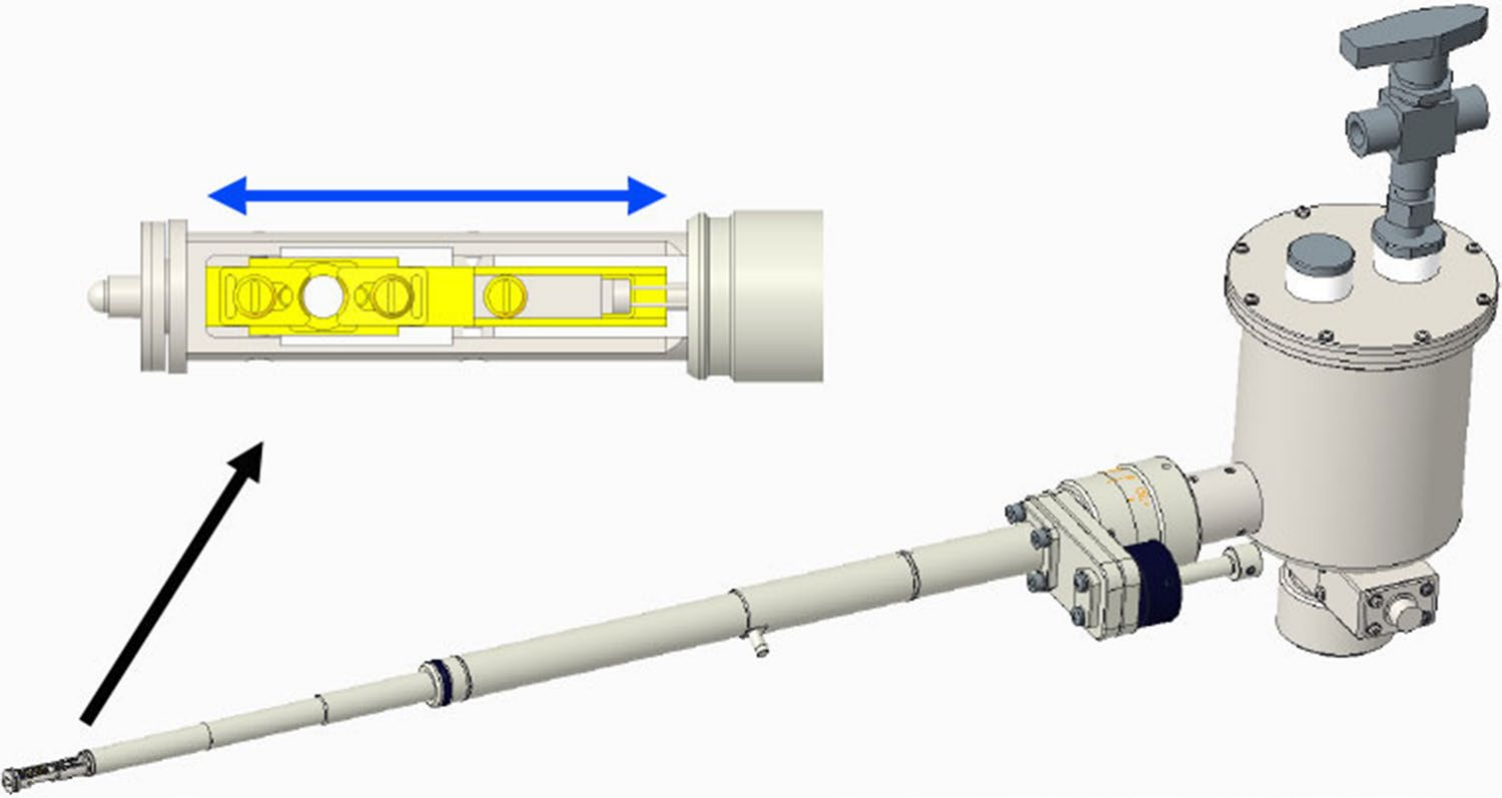
Schematic of the cryo-transfer holder. The inset shows an enlarged view of the specimen stage at the tip. The stage slides into the arm to prevent frost formation during transfer into the specimen chamber of the cryo-TSEM.

**Figure 3:**
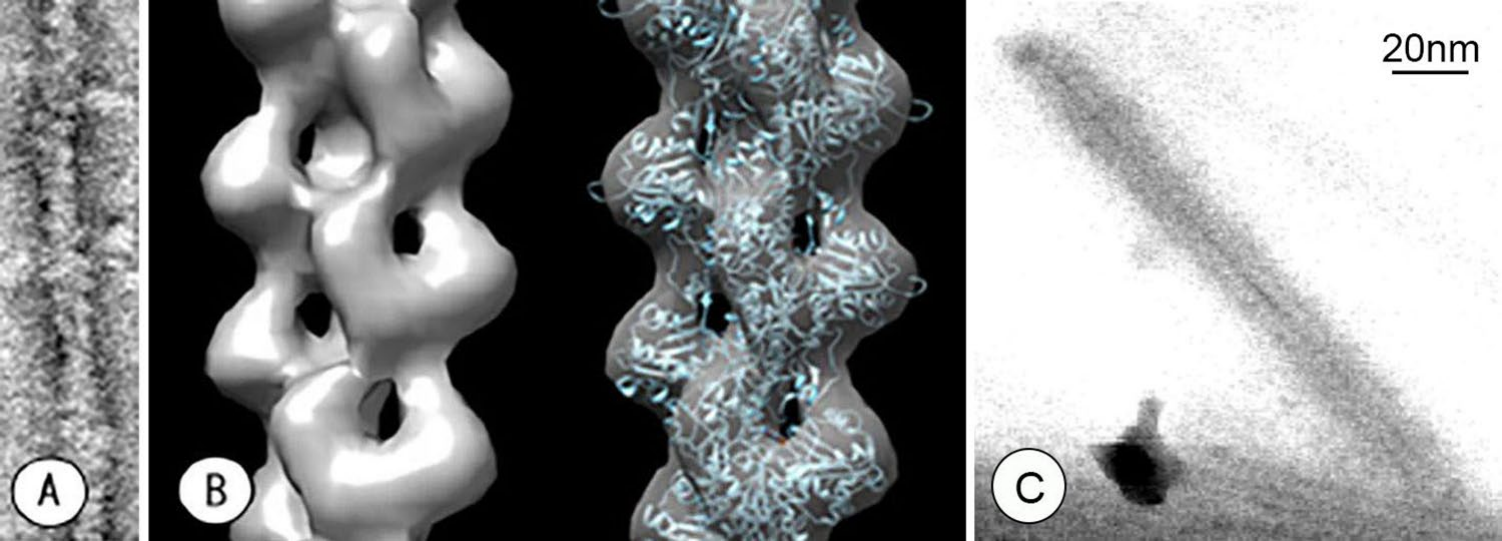
Evaluation of images obtained with STEM optical system installed in the 30-kV TSEM. (**A**) A raw STEM image of negatively stained actin filaments. The short and long periodicities can be observed in high contrast. (**B**) Reconstructed molecular model of actin filaments via image processing of 129 units images (one unit contains approximately 28 monomer conjugates). The molecular mapping^25^ derived by X-ray diffraction was superimposed on the reconstructed image (right). This demonstrates that the STEM images had sufficient resolution for imaging intracellular fine structures. (**C**) A raw STEM image of quickly frozen TMV acquired at – 180 °C. Although this image is slightly affected by sample drift, the 2.3 nm spiral pitch is clearly visible with high contrast.

The cryo-TSEM, including a cryo-transfer holder and anti-contamination trap (both newly developed), functioned as a standard cryo-electron microscope (Supplementary Fig. S1). Both the holder and the trap had double-pipe structures for venting gas; thus, each easily filled their tip regions with liquid nitrogen. The specimen stage of the holder could be cooled to − 190 °C with a liquid nitrogen slush obtained by vacuum pump evaporation (Supplementary Fig. S2). Similarly, the trap could be cooled to − 210 °C by using the slush (Supplementary Fig. S3). The sample temperature during imaging was substantially dependent on that of the trap. If the trap temperature was the same as, or higher, than that of the specimen stage on the holder, then water sublimated from the ice around the sample was not efficiently adsorbed on the trap and was refrozen on the sample surface as frost contamination. To prevent this, the stage temperature had to be set to approximately 20 °C above the trap temperature. Thus, the sample had to be observed above − 180 °C. The water–adsorption capacity of the trap was also very important because many water molecules sublimated, and must be adsorbed, especially when observing cells embedded in large amounts of ice. Therefore, the tip shape of the trap was designed to enclose the specimen stage of the holder, as shown in Fig. 1, because it not only adsorbed sublimated water molecules, but also had a path for multiple electron beams such as transmitted, secondary, and backscattered electrons. The sizes of the upper and lower holes were important for efficient electron paths, as determined by trial and error. The specimen stage of the holder slid into the holder arm to prevent frost formation after placing samples in a cryo-workstation (Supplementary Fig. S4), and was pulled out during imaging (Fig. 2). The cryo-TSEM was based on an in-lens type SEM (Hitachi SU9000), equipped with detectors for transmitted, secondary, and backscattered electrons, and was able to capture three images simultaneously. However, the electron dose was limited to ~ 30 electrons/Å^2^ to protect the frozen cells from irradiation damage; hence, backscattered electrons from the ice surface were too weak to generate an image. A secondary electron image (SEM image) was observed with little charging because of the electron conductivity of the ice. Transmission images were acquired by scanning an extremely thin electron beam emitted from a CFEG (so called STEM images). The diameter of the scanning beam was 0.4 nm, with a spherical aberration coefficient of 2 mm, and optimum opening angle of 11 mrad^29^. Cryo-TSEM (prototype) was developed based on the existing SEM, but several points such as detector sensitivity, vacuum system, and sample chamber were also improved. The quality of the STEM imaging was initially evaluated by using negatively stained actin filaments at room temperature. The short and long periodicities of the actin filaments were observed in a raw image at high contrast (Fig. 3A). STEM images can be also used for three-dimensional reconstruction via single-particle analysis without contrast transfer function (CTF) correction. Reconstruction of an actin filament was performed via single-particle analysis using 129-unit images (one unit contained approximately 28 monomer conjugates). Because the molecular model^30^ was adequately superimposed on the reconstructed image (Fig. 3B), it verified that the STEM system installed in cryo-TSEM provided sufficient resolution for cell observations. These nice properties of STEM system have exhibited even at – 180 °C. As shown in Fig. 3C, in the raw image of tobacco mosaic virus (TMV) embedded in ice, a 2.3 nm spiral pitch of capsid proteins in TMV was clearly observed with high contrast. However, this image appeared to be blurry, when viewed with larger magnification. The blurring is largely due to the sample drift at low temperature. Therefore, our microscope system is yet to be optimized before it could be utilized for single particle analysis to sub-nm resolution. Future development to enable high-resolution analysis at -180℃ would involve the making of a drift-free cryo-transfer holder.

### Application of cryo-TSEM to the analysis of cell structures

If cells are quickly and suitably frozen, the intracellular fine structures will be preserved in a nearly native state. Therefore, it was very important to observe quickly frozen cells for understanding their native structure. With plunge freezing in liquid ethane at − 185 °C, vitreous ice formation was confined to the cortical region of the cell, because the size of the cell and the amount of water surrounding it are large. However, it is also true that even if nanometre-sized ice crystals are formed, they are too small to be observed in images taken at 40,000× magnification or less. Cultured cells are a few microns thick, and are frozen together with a large amount of water. Therefore, it is necessary to reduce their thickness to allow electrons to pass through. In previous works, frozen samples were thinned with a cryo-ultramicrotome (CEMOVIS)^7^, or a focused ion beam^19–22^. By contrast, this study employed unroofing method^23,31–33^ to expose the cytoplasmic surface of the ventral cell membrane for imaging membrane cytoskeletons instead of above methods. In practice, however, not all cells were unroofed in the same way. As a result, unroofed cells varied in thickness and cytoplasmic contents. The various extents of unroofing were conveniently classified into fully unroofed cells, partially unroofed cells, and micro-unroofed cells (see Supplementary Fig. S5). Because specimens were usually covered with thin layer of ice regardless of the degree of unroofing, intracellular structures became more vivid with increasing ice sublimation. To reduce the thickness of the ice layer for electron passage, the temperature of the specimen stage was initially increased to − 100 °C to accelerate ice sublimation, and then decreased to − 180 °C for imaging. It should be noted that the temperature cycling capability was a novel advantage of this cryo-TSEM.

**Figure 4:**
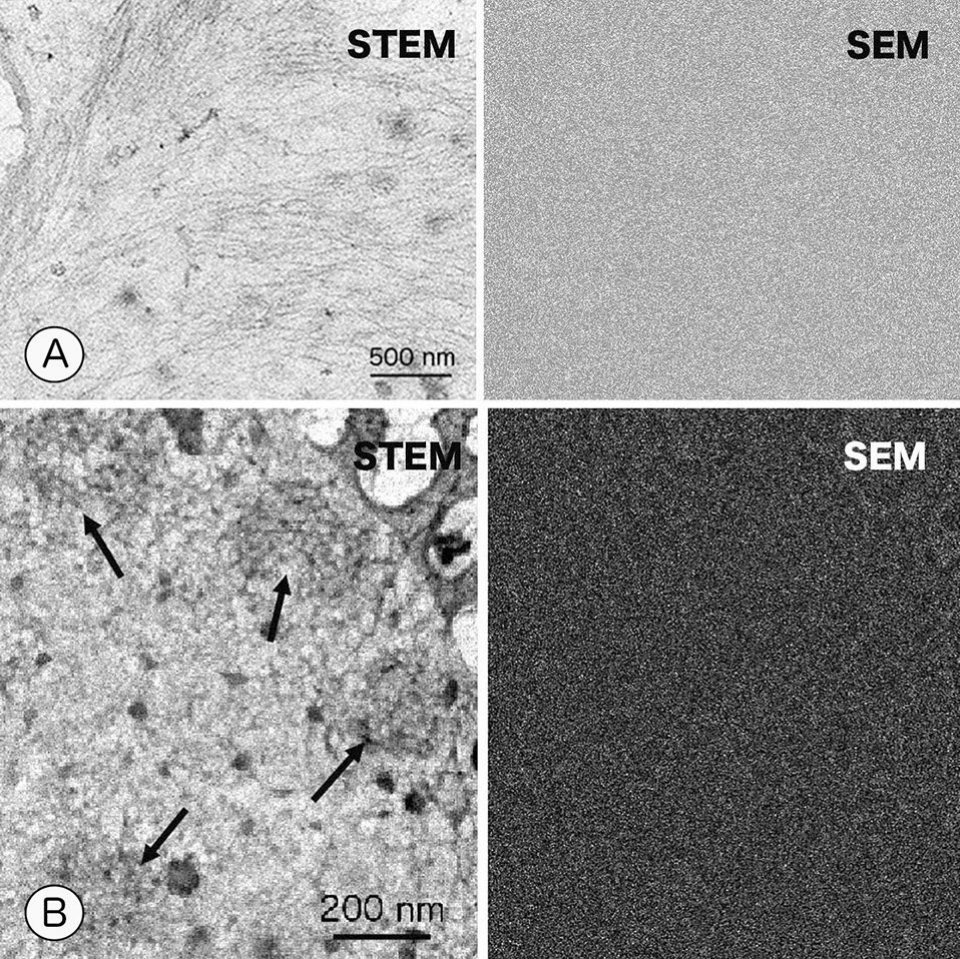
Simultaneous imaging of fully unroofed frozen cells with cryo- STEM and SEM modes. (**A**) Cortical actin filaments attached to the cytoplasmic (inner) surface of a cell membrane. (**B**) Clathrin coats (arrows) on the inner surface of the cell membrane in fully unroofed cells. Fully unroofed cells were thin enough for electrons to pass through. The SEM image appears flat because the cells were completely embedded in ice.

**Figure 5:**
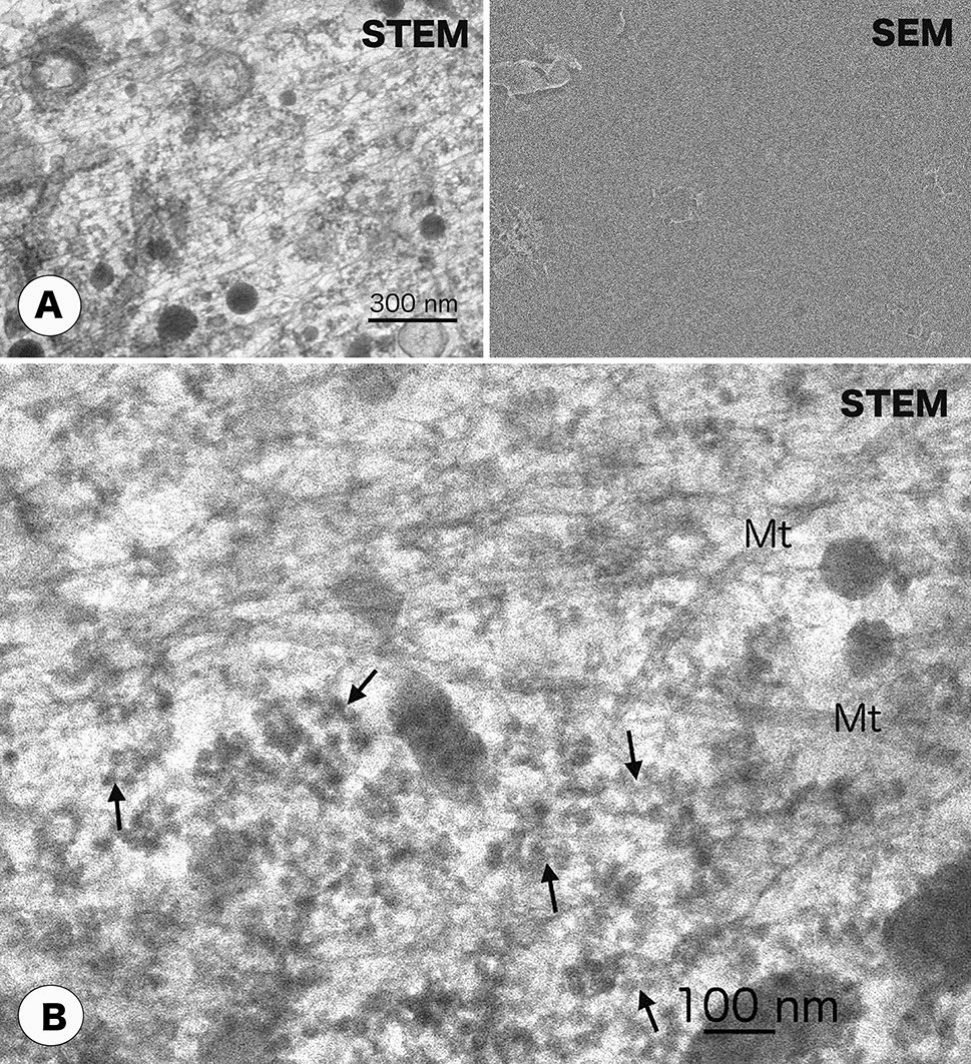
Cryo-electron micrographs of partially unroofed cells. (**A**) Simultaneous measurement by STEM and SEM modes revealed cytoskeletons, ribosomes, and the endoplasmic reticulum. In partially unroofed cells, the cell membrane was removed, but many organelles remained intact. The total thickness of the cell was sufficiently reduced by unroofing so that STEM images could be observed with little ice sublimation. The unroofed cell was completely embedded in ice, as shown by the flatness of SEM image. (**B**) Magnified cryo-STEM micrograph of the same sample shown in Figure (**A**). Poly-ribosomal-chains (arrows) and microtubules (Mt) are clearly observed. Ribosomes are often interconnected by extremely thin threads to form a ribosome chain. Because these structures are observed to overlap with other filaments and membrane structures, this unroofed cell appears to be a little bit thick.

**Figure 6:**
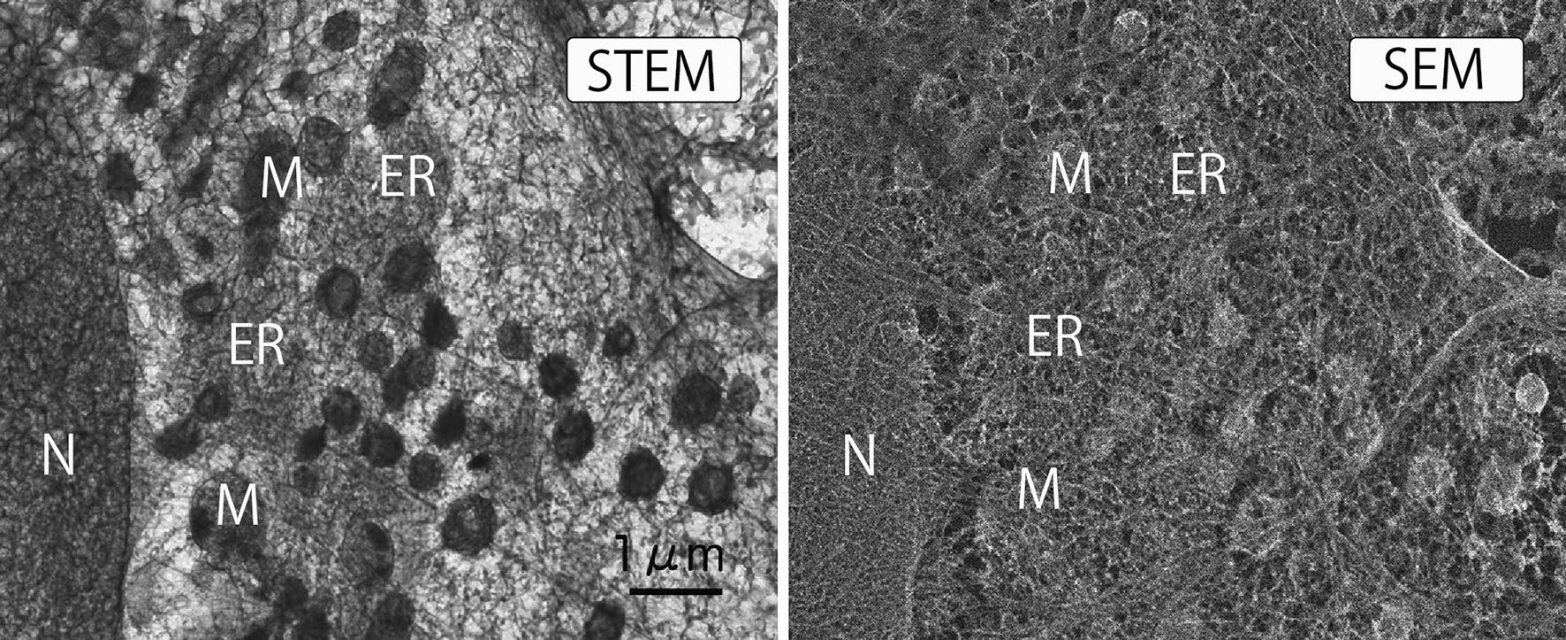
Simultaneous cryo-STEM image and SEM image showing a partially unroofed cell. Many organelles remain despite complete ablation of the apical cell membrane. Deep sublimation reduced the ice layer for electrons to pass through the cell. At the same time, the undulation in the cytoplasm could be observed in the SEM image. *N* nucleus, *M* mitochondria, *ER* endoplasmic reticulum.

In fully unroofed cells with little cytoplasm, the cytoplasmic surface of the ventral membrane was completely exposed, and clathrin coats, cortical actin filaments, and microtubules were found in close contact with the cytoplasmic surface of cell membrane (Fig. 4). Actin filaments and microtubules grew without fragmentation or branching, while bending gently. The total thickness of fully unroofed cells was estimated to be 50 nm or less, and could be observed without sublimating ice. Partially unroofed cells exhibited variations in thickness, ranging approximately 50 nm to 1 micron thick because of residual organelles and cytoplasm, although most of the apical cell membrane was removed. Figure 5 shows partially unroofed cells whose cytoplasm and water were frozen to an appropriate extent. Actin filaments, ribosomes, and the endoplasmic reticulum (ER) appeared after a modest degree of sublimation. These cells were completely embedded in ice even after ice sublimation as the SEM image showed a flat surface. Judging from the overlapping of the fine structures, we estimated the thickness of the unroofed cell to be 150 nm. This far exceeds 30 nm, the presumable mean free path of 30 kV electrons in a uniform, dense sample, such as resin. Nevertheless, unroofed frozen cells of this thickness could be imaged with a 30 kV cryo-TSEM. Cells unroofed to this extent provided the most information about structures. Figure 5B shows a high-magnification image of another region of the same cell shown in Fig. 5A. The ER, ribosomal chains, microtubules, and actin filaments were clearly observed overlapping each other. In another partially unroofed cells containing many organelles, total thickness of cells and ice surrounding them was too thick to be observed without sufficient sublimation of ice. The nucleus, mitochondria, ER and cytoskeletal filaments were revealed after sublimation of ice for 20–30 min at − 100 °C (Fig. 6). Sublimation of ice reduces the thickness of ice layer, but does not change the thickness of cell structure. That is, parts of the structure were exposed from the ice layer, which were detected as SEM images (Fig. 6 right). Thus, simultaneous SEM imaging was useful for investigating the freeze-drying state.

**Figure 7:**
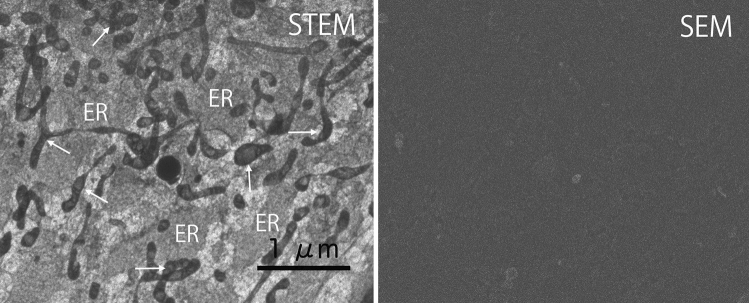
Simultaneous STEM images and SEM image showing a micro-unroofed cell with many organelles. A micro-unroofed cell was similar to a normal cell in appearance, but a very small area of the cell membrane was removed, or very tiny holes were opened. The SEM image did not appear even after ice sublimation more than 1 h because the apical membrane covered most areas of the cell. STEM revealed the spatial architecture of several organelles and filaments, as in high-resolution light microscopy. A large endoplasmic reticulum (ER) network was observed beneath the cell membrane. Mitochondria were also observed, sometimes branching or overlapping with the ER.

**Figure 8:**
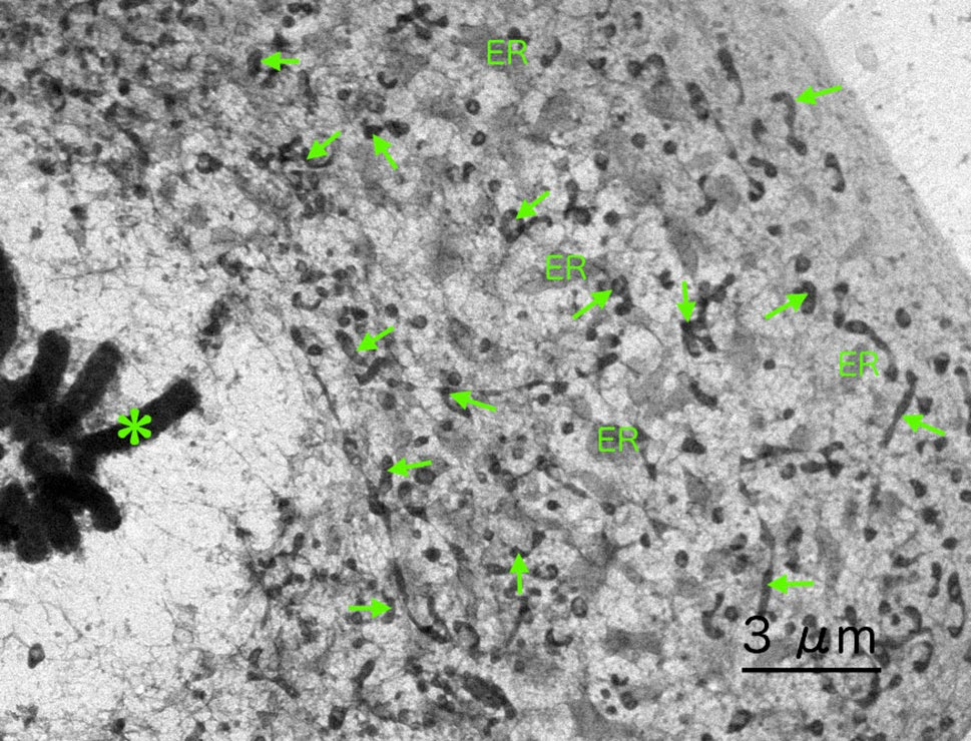
Cryo-electron micrograph of micro-unroofed cells in mitotic telophase. The arrows indicate mitochondria. ER indicates the endoplasmic reticulum. The asterisk shows chromosomes in telophase. Large networks of the ER were preserved well even in mitotic phase despite the disappearance of the nuclear envelope.

On the other hand, micro-unroofed cells appeared to be almost whole without any removal of cell membrane. Therefore, they were too thick for electrons to pass through. Surprisingly, however, the intracellular fine structure of micro-unroofed cells became revealed after prolonged (deep) sublimation of ice despite the very short mean free path of 30 kV electrons (Fig. 7). Unlike Fig. 6, the flat image in SEM mode after deep sublimation indicated that the cell was almost entirely covered with a membrane. Nevertheless, the mitochondria, ER, and cytoskeleton were visible after deep sublimation. Many flat ERs were located beneath the apical cell membrane and were interconnected to form a network (Fig. 7). These networks were also well-preserved in mitotic telophase cells, as shown in Fig. 8. More recently, the spatial ER structure in the mitotic phase was obtained via serial block-face SEM^34^. This observation of thick cells via deep ice sublimation demonstrates another advantage of this cryo-TSEM.

## Discussion

The cryo-TSEM was able to acquire both a STEM and SEM image of unfixed frozen cells simultaneously. Effective simultaneous capture of the image of unfixed cells was possible only in the frozen state. The high electrical conductivity of both the frozen cells and the carbon-coated grids allowed SEM images to be acquired while suppressing sample surface charging. However, the intensity of electrons back-scattered from the frozen sample surface was too weak to generate images as the total number of irradiating electrons was small for mitigating radiation damage. Cold FEG installed in this cryo-TSEM generated extremely thin electron beam with an irradiation angle of 11 mrad (a beam diameter of 0.4 nm), that produced high-contrast STEM images without using phase contrast (Figs. 4 and 5). As a result, CTF correction was not necessary for high-resolution single-particle analysis. Along this line, this cryo-TSEM can be utilized for pre-screening in the workflow of single-particle analysis. As mentioned above, the image quality deteriorated at − 180 °C because of the movement of the specimen stage at low temperature. The direction of the drift was uncertain and the magnitude could not be quantified. It was most likely caused by bubbling generated in the liquid nitrogen slush mixed with liquid nitrogen, while the details demand further investigation. The images appeared to be slightly affected by the drift, but less significantly when they were taken with magnification less than 40,000×. The maximum resolution that could be offered was 0.4 nm when there is no drift. In the future, near-atomic resolution for single-particle analysis might be achieved as the electron beam is made thinner and the stage drift is removed. However, reduction of the drift is closely coupled with the scanning speed and the detector sensitivity. The scanning speed of SEM has hardly changed in the last 50 years, and image acquisition takes approximately 20 to 30 s per frame. Here, 2560 × 1920-pixel images were recorded at 32 s/frame, and 1280 × 960-pixel images were recorded at 16 s/frame. The higher detector sensitivity enabled faster imaging. Therefore, it will be necessary to improve the detector sensitivity and the scanning speed in the future. The adverse effect of drift on imaging would be eliminated if both are sufficiently high and the image could be captured at a greater speed.

As mentioned above, the amount of remaining cytoplasm varied among unroofed cells. In fully unroofed cells consisting of ventral cell membrane and membrane cytoskeletons, the total specimen thickness was approximately 10 to 50 nm, which was thin enough for electrons accelerated at 30 kV to pass through, even if it is completely embedded in ice. The issue was with partially unroofed cells and micro-unroofed cells whose total thickness reached a few microns because many organelles remained in the cytoplasm. The samples were not thin enough to allow electrons to pass through at an acceleration voltage of 30 kV. This fact raises the question of why the structures can be observed simply by sublimating a large amount of ice. Even though cells appeared to be whole, somewhere in the cell membrane seemed to be torn in micron to nanometer size. In this case, the cell fluid (cell sap) was diluted with an influx of external buffer and an outflow of cell fluid through the ruptured areas, or micron-sized holes, in the cell membrane. That is, the cell fluid was replaced partially with buffer solution. Although the normal frozen cell fluid barely sublimated, even under high vacuum, the frozen diluted cell fluid was sublimated by increasing the sample temperature to − 100 °C under a 5 × 10^–6^ Pa vacuum for at least 30 min or more. The deep sublimation of ice substantially reduced the thickness of frozen cell fluid. Therefore, the cells were filled with cytoskeleton, organelles, and voids. In TSEM with STEM optics, the images were formed with amplitude contrast based on the counts of electrons passing through the organelles, cytoskeleton, and the voids. The thickness of ice remaining at the cell bottom after sublimation seemed to be comparable to the mean-free path. As a result, deep sublimation allowed the electron beam to pass through. This is why thick-cell structures emerged after deep sublimation. Thick cells could not be observed without ice sublimation, if they were partially unroofed.

Prolonged sublimation of ice surrounding frozen cells had previously been thought to cause artefacts. Since purified proteins lose their structures with slight drying, raising the sample temperature has been severely restricted to maintain atomic resolution. Therefore, increasing the temperature to accelerate sublimation has not been reported, except for freeze-etching replica electron microscopy. However, no artefacts have yet been detected on images taken at 40,000× magnification. When ice is sublimated in a vacuum, minerals in the buffer should precipitate on the surfaces of organelles and filaments. However, the amount of precipitation was too small to be detected at 40,000× or less. Since fine structures of unfixed frozen cells collapsed on drying completely, the fine structures of unroofed cells should be supported by an ice layer. The organelles that were completely exposed may have been covered with a thin ice layer containing buffer minerals because SEM images were obtained without surface charging. These thin ice layers appeared to maintain the structures of unfixed cells.

In TEM, the electron mean-free path increases with the electron energy via the acceleration voltage. That is, the mean free path is proportional to the acceleration voltage; besides, it also depends on the state of the sample. The mean free path of the 30 kV acceleration voltage was estimated to be approximately 30 nm for a uniform and dense material. This makes imaging of thick cells impossible. However, the mean-free path is just a measure and should be long in biological specimens consisting of light elements. Especially, cells are not a uniform and dense, and will differ greatly depending on whether they are embedded in resin or ice, or whether they are ice-embedded after the cell fluid is removed after unroofing. Therefore, it is difficult to precisely estimate the mean free path in unroofed cells. After all, it is that whether or not the structure can be observed matters.

## Methods

### Cryo- transmission scanning electron microscope (Cryo-TSEM)

An in-lens-type scanning electron microscope (Hitachi SU9000; accelerating voltage, 30 kV), equipped with detectors for transmitted electrons, secondary electrons, and back-scattered electrons was used. The cryo-TSEM was constructed by installing an anti-contamination trap (Fig. 1) and a cantilever-type cryo-transfer holder (Fig. 2) to SU9000 SEM. In addition, sensitivity of detector for transmitted electrons was improved. The trap prevented refreezing of sublimated water as frost on the specimen surfaces. After mounting the frozen cells onto the specimen stage of the cryo-transfer holder in liquid nitrogen, with the assistance of a cryo-workstation (Supplementary Fig. S3), the stage was slid into the holder to prevent frost (Fig. 2). Then, the holder was placed in the specimen chamber of the cryo-TSEM. This was performed for all samples. Liquid nitrogen in the dewar of the holder and trap was evaporated with a vacuum pump to generate nitrogen slush. The pumping was stopped during imaging to prevent vibrations. The specimen temperature was then maintained at − 180 °C for 40 min for imaging. If the sample temperature rose, the imaging was temporarily halted and evaporation was resumed for approximately 5 min to restore the nitrogen slush.

### Sample preparation for cryo-EM

Normal rat kidney cells, purchased from ATCC, USA, were cultured on C-flat gold mesh grids (#200 multi-hole), or molybdenum mesh grids (#200) covered with carbon-coated Formvar (polyvinyl formal), for 1–2 days in a CO_2_ incubator. The culture medium was DMEM (Sigma-Aldrich Co., St. Louis, MO, USA), supplemented with 10% bovine serum. The sonication unroofing method was used to image the membrane cytoskeletons. This also helped to prepare thin cells for quick freezing and to allow electron beams to pass through. Cells cultured on the grids were sequentially washed with Ringer’s solution consisting of 155-mM NaCl, 3-mM KCl, 2-mM CaCl_2_, 1-mM MgCl_2_, 3-mM NaH_2_PO_4_, and 10-mM glucose in 5-mM HEPES buffer (pH 7.4), followed by Ca^2+^-free Ringer’s solution (no CaCl_2_) for a few seconds. The cells were soaked for approximately 10 s in a polylysine solution (Mw 30,000–50,000; Sigma-Aldrich, St. Louis, MO, USA; 0.5 mg/ml dissolved in Ca^2+^-free Ringer’s solution), and then washed three times for a few seconds each in KHMgE buffer consisting of 30-mM HEPES (pH 7.4), 70-mM KCl, 3-mM MgCl_2_, and 1-mM EGTA. Immediately after washing, the cells were unroofed via gentle sonication (0.5 W, 27 kHz) in an isotonic buffer containing 30-mM HEPES (pH 7.4), 70-mM KCl, 3-mM MgCl_2_, 1-mM EGTA, 1-mM DTT and Pefabloc SC (Roche Diagnostics Gmbh, Mannheim Germany) protease inhibitor. The unroofed cells were washed briefly in the same fresh buffer. See previous reports for details^31–33^.

### Quick freezing

Unroofed cells were immediately frozen with a Leica EM-GP quick freezer (Leica microsystems GmbH, Vienna, Austria). Excess water surrounding the unroofed cells was automatically absorbed, on only the grid side, with filter paper for 5–6 s. Then, the samples were automatically plunged into liquid ethane cooled at − 185 °C with liquid nitrogen. Frozen samples were stored temporarily in liquid nitrogen.

For tobacco mosaic virus (TMV)(Fig. 3C), Quantifoil multi hole Cu 200 mesh grids were mounted in Leica EM GP quick freezer. Subsequently, 2.0 μl of approximately 1 mg/ml TMV solution, purchased from ATCC, USA, was applied onto the grids, and then frozen in the same way.

### Ice sublimation

Ice sublimation was performed by increasing the temperature to − 100 °C under high vacuum (5 × 10^–6^ Pa) in the cryo-TSEM. The ice thickness was estimated from a low-magnification SEM mesh image. Upon reaching an appropriate ice thickness, the temperature was lowered to − 180 °C for imaging.

### Image processing

Image processing with was performed EOS software^35^ to evaluate the image quality of negatively stained actin filaments.

For STEM, the display magnification was 120,000×. Slow scans of 16 s for 1280 × 960-pixel images were captured at 0.827 nm/pixel. Three-dimensional reconstruction was performed without CTF correction. Approximately 28 actin monomer conjugates were used as one unit for single-particle analysis. An actin filament was reconstructed from 129 units by using a spiral symmetry. Details of actin purification and the single-particle analysis have been previously described^36–38^.

## Supplementary Information

Supplementary Figures.
