# Supplementary material for: A cryo-TSEM with temperature cycling capability allows deep sublimation of ice to uncover fine structures in thick cells
Source: Sci Rep. 2021 Nov 1;11:21406. doi: 10.1038/s41598-021-00979-z (PMC8560947; doi:10.1038/s41598-021-00979-z)
Supplement: Supplementary file 1 — Supplementary Figures. [file 41598_2021_979_MOESM1_ESM.pdf]

A cryo-TSEM with temperature cycling capability allows deep sublimation of ice to uncover fine structures in thick cells

SUPPLEMENTS

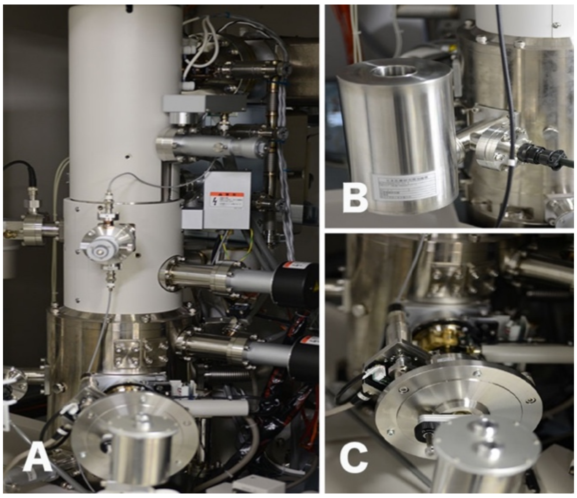

Fig. S1. Overview of the prototype of a cryo-S(T)EM instrument integrated with elemental technologies.
A: Front view of the cryo-S(T)EM prototype microscope column
B: Left-side view of the anti-contamination trap installed on the column
C: Front view of the cantilever type cryo-transfer holder assembled into pre-evacuation chamber. The pre-evacuation chamber was kept dry by leaking with nitrogen gas.

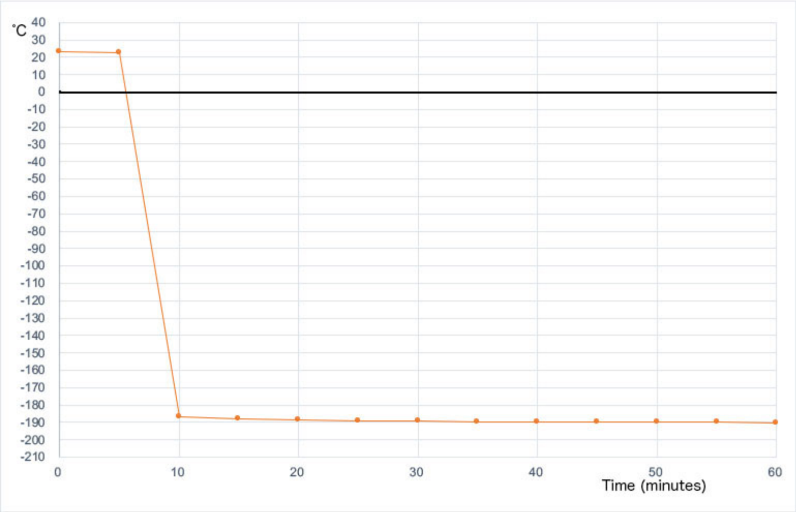

Fig. S2. Diagram of changes in the temperature of the sample stage of the cryotransfer holder immediately after injecting liquid nitrogen into the Dewar. Evaporation to generate a liquid nitrogen slush started at approximately -180 °C. When the temperature reached at -190 °C, evaporation was continued for another 3-5 minutes and then stopped.

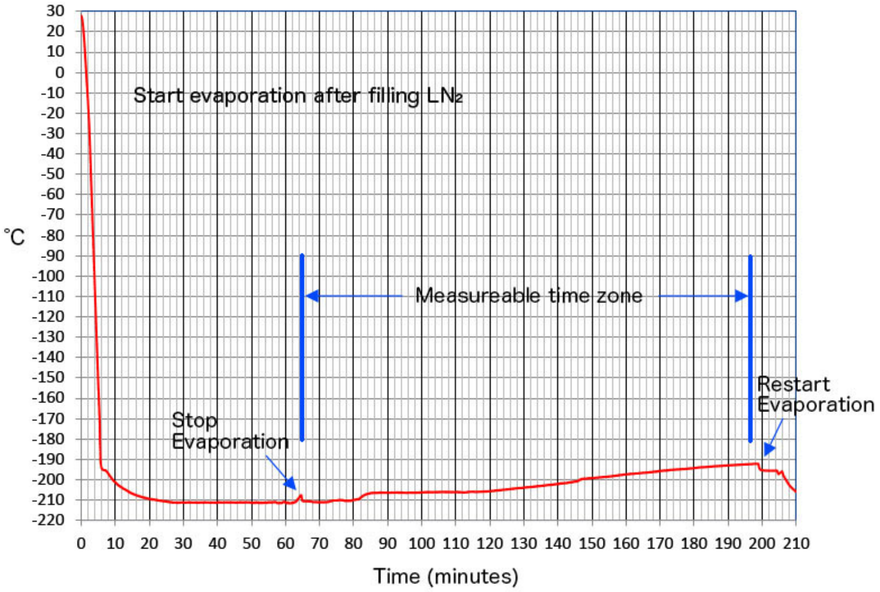

Fig. S3. Diagram of changes in the temperature of the tip area of the anti-contamination trap after injecting liquid nitrogen into the Dewar. The temperature was maintained below -190 °C for approximately 40 minutes after stopping evaporation to make nitrogen slush.

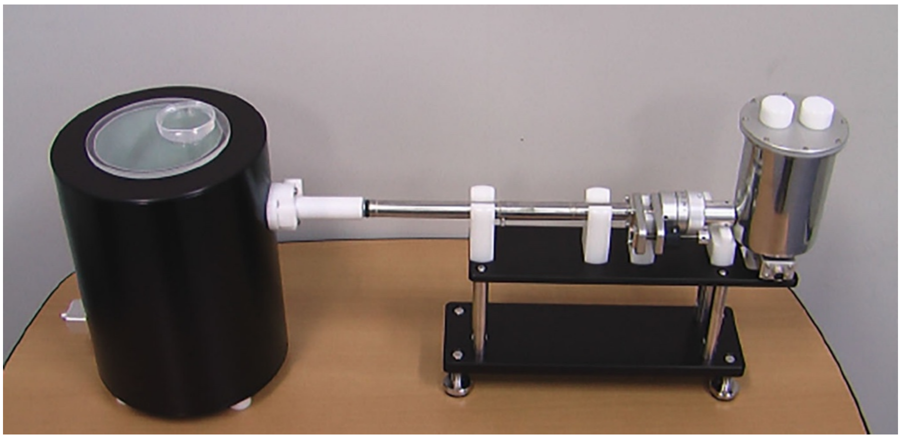

Fig. S4. Prototype cryo-station assembled with a cryo-transfer holder.
The cryo-station was used for mounting samples onto the specimen stage of the cryo-transfer holder in liquid nitrogen.

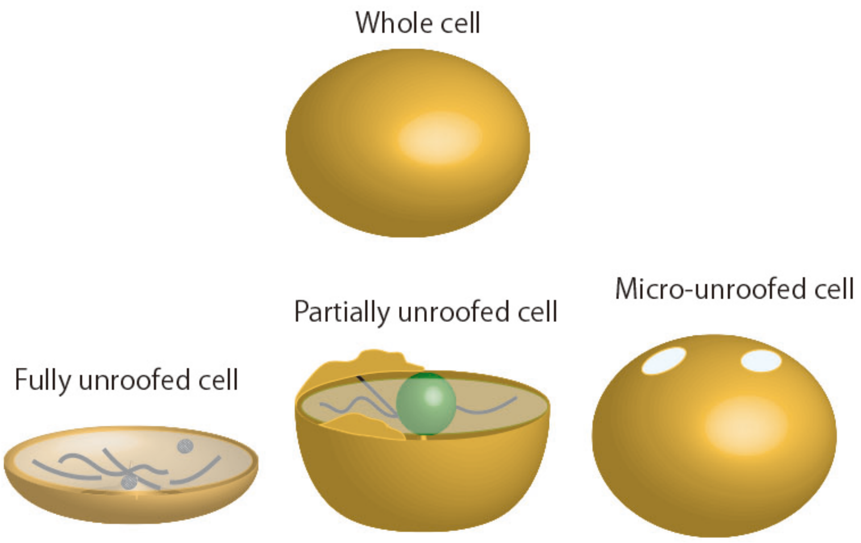

Fig. S5 Illustration showing degree of unroofing. All cultured cells are not uniformly unroofed, and the degree of unroofing varies from cell to cell. In this study, the observation results are described according to the three categories shown in the above figure for convenience.
